# The Story of the Bacteria and Their Relations to Health and Disease

**Published:** 1891-05

**Authors:** 


THE STORY OF THE BACTERIA AND THEIR RELATIONS IN HEALTH AND DISEASE. By T. MITCHELL PRUDDEN. 76mo, p. 143. New York and London: G. P. Putnam's Sons. The Knickerbocker Press. 1891. Price, 75c.

Within the past few years the study of the bacteria has made incomparable strides and their characteristics and pathogenic virtues have been carefully and diligently noted. The general practitioner and the scientifically inclined physician have not had the opportunity or time to look into the subject carefully, and the larger works on this subject have been ignored. This little book is intended for just this class of readers—taking up the subject in a popular and persuasive manner, being careful to preserve simplicity and intelligibility. The book is further enhanced by the many hygienic points, which the author frequently throws out in connection with the various pathogenic organisms.

The typography is neatly and carefully executed, and is similar to the author's "Dust and Its Dangers." W. C. K.

